# Two new species of crayfish of the genus *Cherax* from Indonesian New Guinea (Crustacea, Decapoda, Parastacidae)

**DOI:** 10.3897/zookeys.769.26095

**Published:** 2018-06-26

**Authors:** Christian Lukhaup, Rury Eprilurahman, Thomas von Rintelen

**Affiliations:** 1 Waldstrasse 5a, 66999 Hinterweidenthal, Germany; 2 Animal Systematics Laboratory, Faculty of Biology, Universitas Gadjah Mada Jl. Teknika Selatan, Sekip Utara Yogyakarta 55281, Indonesia; 3 Museum für Naturkunde – Leibniz Institute for Evolution and Biodiversity Science, Invalidenstraße 43, 10115 Berlin, Germany

**Keywords:** freshwater, morphology, molecular phylogeny, New Guinea, taxonomy

## Abstract

Two new species of the genus *Cherax* are described and illustrated. *Cherax mosessalossa* **sp. n.**, endemic to the Klademak Creek drainage in Sorong, in the western part of the Kepala Burung (Vogelkop) peninsula, West Papua, Indonesia, is described, figured and compared with its closest relatives, *Cherax misolicus* Holthuis, 1949 and *Cherax warsamsonicus*. The new species may be easily distinguished from both by the shape of the rostrum, the shape of the chelae, the presence of five cervical spines, the shape of the scaphocerite, and short scattered hairs on the carapace. *Cherax alyciae* **sp. n.**, endemic to creeks in the Digul River drainage in the eastern part of the Boven Digoel Regency, Papua, Indonesia, is described, figured, and compared with its closest relative, *Cherax peknyi* Lukhaup & Herbert, 2008. The new species may be easily distinguished from *Cherax peknyi* by the shape of the chelae, presence of a soft patch on the chelae of the males, and colouration. A molecular phylogeny based on two mitochondrial gene fragments, 16S and COI, supports the morphology-based description of the two new species, which can also be clearly distinguished by sequence differences.

## Introduction

The crayfishes of the island of New Guinea were extensively studied by Holthuis (1949, 1956, 1958, 1982, 1986, 1996), with additions by Lukhaup and Pekny (2006, 2008a), Lukhaup and Herbert (2008), Lukhaup (2015), Lukhaup et al. (2015), Patoka et al. (2015), Lukhaup et al. (2017) and Patoka et. al (2017).

In January 2016 the first author visited Sorong Regency and South Sorong Regency, West Papua, Indonesia, to clarify the distribution of some crayfish species present in the pet trade. During the stay in Sorong, we also had the chance to visit a creek at the edge of the city where our guide, Marten Luter Salossa, showed us a *Cherax* species. In the present contribution, this species is described as new to science. *Cherax mosessalossa* sp. n. is genetically and morphologically most similar to *Cherax misolicus* Holthuis, 1949 endemic to the Island of Misool, one of four major islands in the Raja Ampat Islands in West Papua, Indonesia and to *Cherax warsamsonicus* endemic to the Warsamson River drainage, in the western part of the Kepala Burung (Vogelkop) peninsula.

*Cherax mosessalossa* sp. n. may be easily distinguished from both by using sequence divergence, by colouration and pattern of live individuals, by the shape of the chelae, the shape of rostrum, and presence of setae on the carapace in *C. mosessalossa* which is absent in *C. misolicus* and *C. warsamsonicus*. *Cherax alyciae* sp. n. is genetically and morphologically most similar to *Cherax peknyi* Lukhaup & Herbert, 2008, from Tamu Creek, in the Fly River drainage, Papua New Guinea. *Cherax alyciae* sp. n. may be easily distinguished from *Cherax peknyi* by colouration and pattern of live individuals, by the shape of the chelae and a soft patch present on the chelae of the males, the shape of rostrum, fewer setae on the ventral side of the chelae, and by using sequence divergence.

## Materials and methods

Samples of *Cherax mosessalossa* sp. n., *Cherax alyciae* sp. n., and *C. peknyi* were collected from creeks in West Papua and Papua provinces (Table 1). Holotypes and allotypes were photographed and kept alive in indoor tanks until samples were obtained for DNA analysis. After this procedure, the animals were preserved in 70% ethanol. Morphometric parameters of all individuals were taken using an electronic digital caliper with an accuracy of 0.1 mm. For the molecular analyses, sequences from an additional ten species of *Cherax* and from two other parastacid genera used as outgroup were downloaded from GenBank (see Table 1).

**Table 1.** Material studied with GenBank accession numbers. Sequences of species represented by more than one sequence are listed consecutively as labelled in Figure 19.

Species/sample	Location	GenBank acc. nos	
		COI	16S
*C. boesemani*	Ajamaru Lake, Papua Barat; 1°17'19.97"S, 132°14'49.14"E; January 23, 2016	KY654084 KY654085	KY654089 KY654090
*C. alyciae* sp. n.	Unnamed creek, Boven Digoel Regency, West Papua, Indonesia; December 7, 2016	MH457597 MH457599 MH457598	MH457588 MH457590 MH457589
*C. communis*	Lake Paniai	–	MH457602
*C. gherardiae*	Pet trade	KU821417	KU821417
*C. holthuisi*	Papua Barat	KU821419	KU821433
*C. misolicus*	Misool Island, South of Papua Barat (Leiden Museum)	–	KJ920813
*C. monticola*	Baliem River, Wamena, Papua	KF649851 –	KF649851 KJ920818
*C. mosessalossa* sp. n.	Klademak Creek, Sorong City 0°52'23.59"S, 131°16'24.40"E; January 26, 2016	MH457602 MH457602	MH457594 MH457595
*C. paniaicus*	Lake Tage, Papua (Field collection)	KJ950528	KJ920830
*C. peknyi*	Unnamed Creek, tributary of Fly River, Papua New Guinea	MH457600 MH457601 MH457604	MH457591 MH457592 MH457596
*C. pulcher*	Hoa Creek (Teminabuan), Papua Barat; 1°28'32.73"S, 132°3'54.94"E; January 23, 2016	KY654083	KY654088
*C. snowden*	Oinsok (Ainsok River Drainage), Papua Barat; 1°11'40.07"S, 131°50'1.14"E; January 24, 2016	KY654082	KY654087
*C. warsamsonicus*	Small tributary to Warsamson River; 0°49'16.62"S, 131°23'3.34"E; January 20, 2016	KY654086	KY654091
	Papua Barat	KU821424 KU821426	KU821438 KU821437
*Engaeus strictifrons*	Crawford River, Victoria, Australia	AF493633	AF492812
*Euastacus bispinosus*	Crawford River, Victoria, Australia	AF493634	AF492813

All material has been deposited at the Museum Zoologicum Bogoriense (= Bidang Zoologi) Research Centre for Biology (= Pusat Penelitian Biologi), Indonesian Institute of Sciences (= LIPI), Jalan Raya Jakarta-Bogor Km 46 Cibinong 16911, Indonesia.

DNA was purified from approximately 2 mm³ of muscle tissue with a Qiagen BioSprint 96 using the manufacturer’s protocol. Polymerase chain reaction (PCR) was used to amplify two mitochondrial gene fragments, a ~535 bp region of the 16S ribosomal RNA gene (16S) using primers 1471 and 1472 (Crandall and Fitzpatrick 1996) and a 710 bp fragment of the Cytochrome Oxidase subunit I gene (COI) using primers LCO1490 and HCO2198 (Folmer et al. 1994).

PCR was performed in 25 µl volumes containing 1x Taq buffer, 1.5 mM MgCl2, 200 µM each dNTP, 1 U Taq polymerase, ca. 50-100 ng DNA and ddH2O. After an initial denaturation step of 3 min at 94 °C, cycling conditions were 35 cycles at 94 °C for 35 s, 45 °C (COI) or 50 °C (16S) for 60 s, and 72 °C for 1 min (COI) or 90 s (16S), with a final elongation step of 5 min at 72 °C. The same primers were used in PCR and sequencing. PCR products were sent to Macrogen Europe for purification and cycle sequencing of both strands of each gene.

Sequences were aligned by eye (COI) and with MAFFT (16S) using the G-INS-i strategy suitable for thorough alignments of sequences with global homology (Katoh et al. 2002). The resulting alignments had a length of 658 bp (COI) and 542 bp (16S), respectively. To determine the best substitution model for Bayesian information analyses (see below), hierarchical likelihood ratio tests were carried out with jModelTest (Posada 2008) on both sequence sets (24 models tested). Based on the Akaike Information Criterion and the Bayesian Inference Criterion, the GTR + I + G (COI) (BIC: HKY + I + G; more complex model chosen) and the HKY + G (16S) models were chosen. The two sequence datasets were subsequently analysed both separately and combined.

Phylogenetic trees were reconstructed by maximum parsimony (MP) using the heuristic search algorithm as implemented in PAUP* (Swofford 2002), with gaps treated as fifth base. Support for nodes was estimated by bootstrap analysis (1,000 bootstrap replicates with 10 random addition sequence replicates each). Maximum Likelihood (ML) analyses were conducted with RAxML (Stamatakis et al. 2008; RAxML BlackBox; 100 bootstrap replicates) under the GTR + (I) + G model of sequence evolution. In addition, Bayesian inference was employed to infer phylogeny by using MrBayes 3.2.2 (Ronquist and Huelsenbeck 2003). The MCMCMC-algorithm was run with four independent chains for 10,000,000 generations, samplefreq = 500, and burnin = 10,001) using the models specified above. The combined dataset was subjected to a partitioned analysis (ML and BI) using the different models for the two genes in the BI analyses. Genetic distances were calculated using MEGA 7.0 (Kumar et al. 2016.). All new sequences have been deposited in GenBank (see Table 1).

## Systematics

### Parastacidae Huxley, 1879

#### Genus *Cherax* Erichson, 1846

##### Cherax mosessalossa sp. n.

Taxon classificationAnimaliaDecapodaParastacidae

http://zoobank.org/B14DD6CD-7368-451A-A91A-4C97F0F8F54A

**Figure 1.**
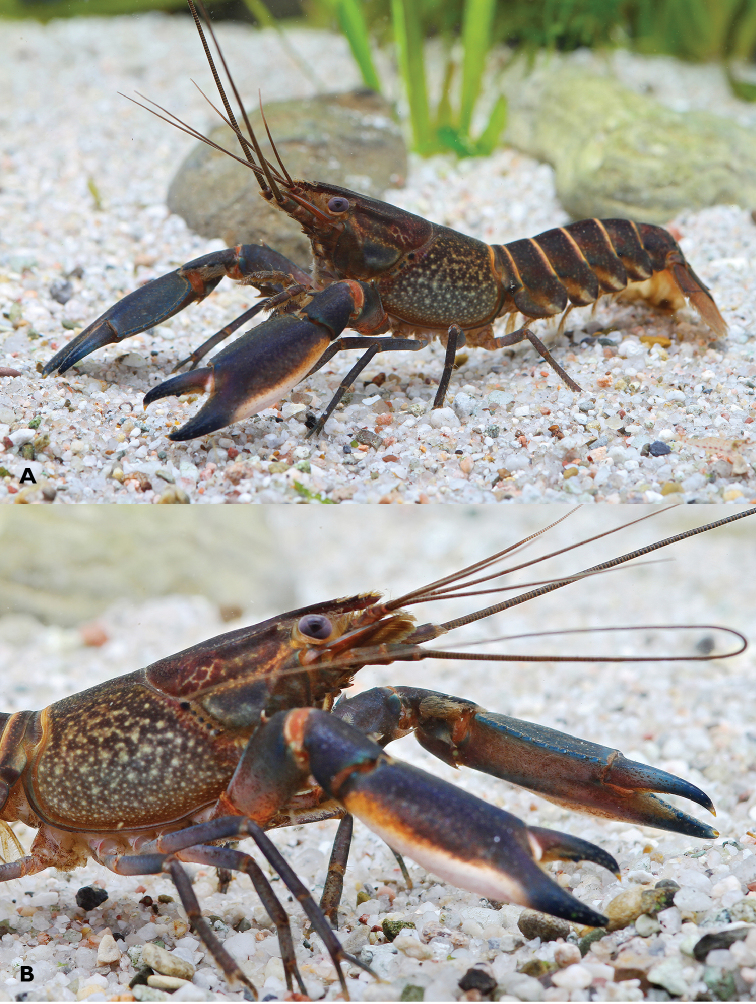
*Cherax mosessalossa* sp. n. **A** holotype male (MZB Cru 4675) from Klademak Creek, Sorong City **B** idem, side view.

**Figure 2.**
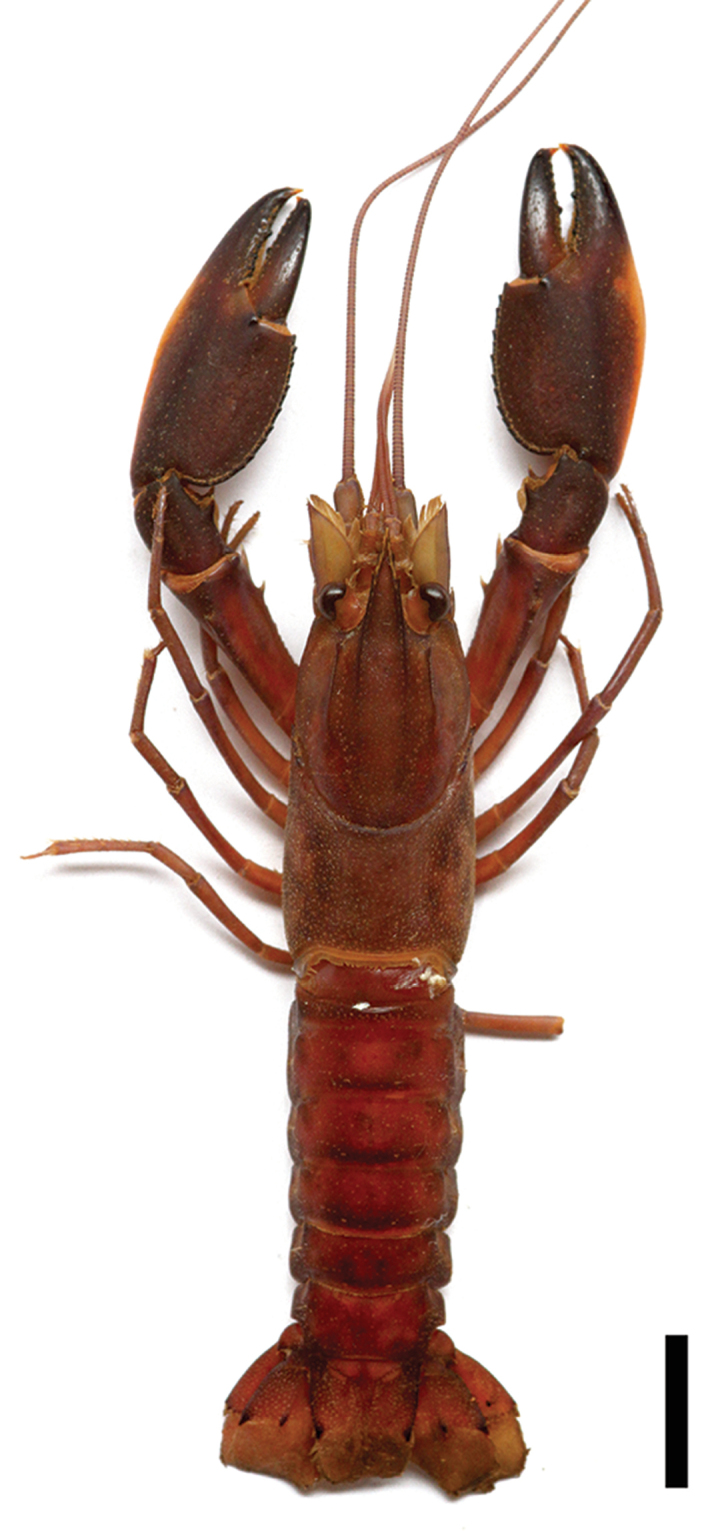
*Cherax mosessalossa* sp. n. holotype male (MZB Cru 4675). Scale bar: 10 mm.

**Figure 3.**
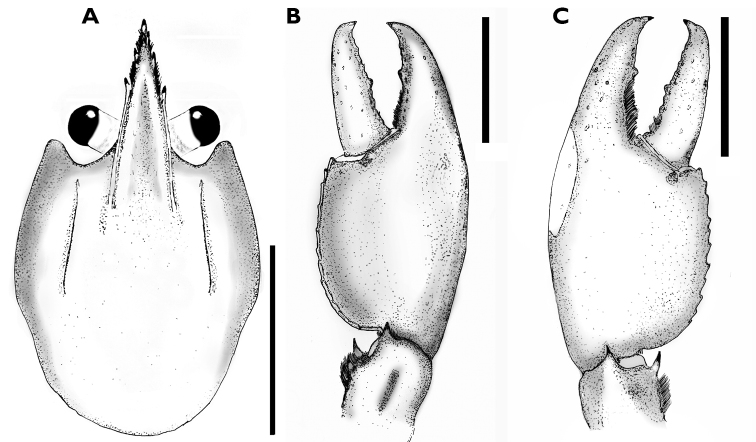
*Cherax mosessalossa* sp. n. holotype male (MZB Cru 4675). **A** dorsal view of carapace **B** dorsal view of right chelae **C** ventral view of right chelae. Scale bars: 10 mm.

**Figure 4.**
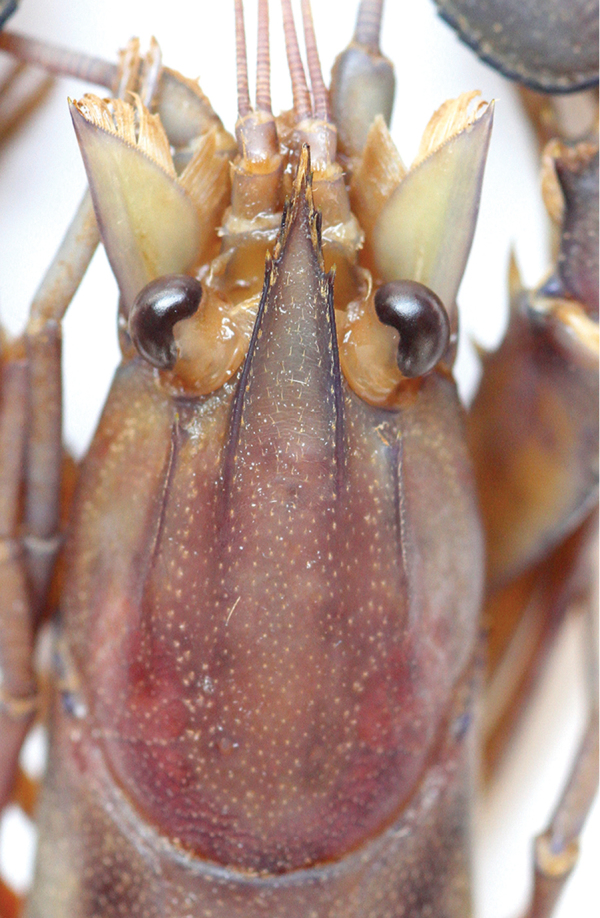
*Cherax mosessalossa* sp. n. holotype male (MZB Cru 4675), dorsal view of cephalothorax. Scale bar: 10 mm.

**Figure 5.**
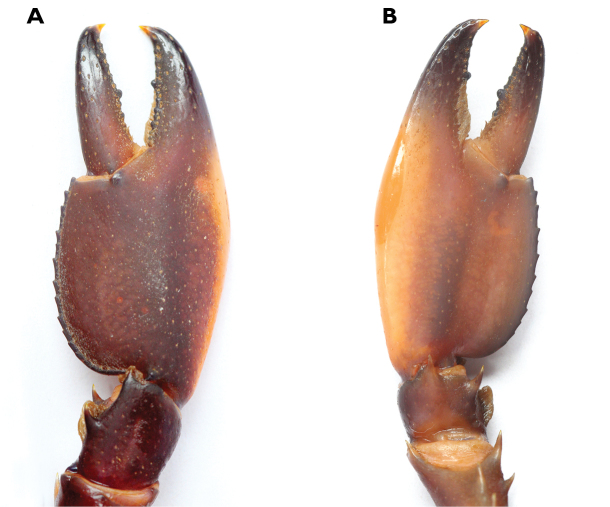
*Cherax mosessalossa* sp. n. holotype male (MZB Cru 4675) . **A** right first chela, dorsal aspect **B** right first chela, ventral aspect. Scale bars: 10 mm.

Figs 1 , 2 , 3 , 4 , 5

###### Material examined.

**Holotype**: male (MZB Cru 4675), under rocks and among roots along banks of Klademak Creek, Sorong City, 0°52'23.59"S, 131°16'24.40"E, West Papua, Indonesia, coll. Christian Lukhaup, Marten Luter Salossa and Salvatore a`Paulo Narahawarin, January 26, 2016. **Allotype**: female (MZB Cru 4676), same data as holotype. **Paratypes**: (MZB Cru 4677), same data as holotype.

###### Diagnosis.

Carapace surface smooth with scattered fine hairs, five small spiniform tubercles posterior to cervical groove on lateral carapace. Eyes large, pigmented. Cornea slightly broader than eyestalk. Rostrum triangular in shape with elevated margins, setose in the anterior half. Rostral margins with three prominent teeth. Rostral carinae prominent. Postorbital ridges prominent with one acute tubercle at anterior terminus. Uncalcified patch on lateral margin of chelae of adult male white, translucent. Propodal cutting edge with row of small granules and one larger tubercle. Chelipeds blue-grey lateral margins white, posterior lateral part sometimes orange. Fingers dark black blue with hooked yellow tips. Other walking legs blue-gray. Pleon blue-grey with yellow transverse lines.

###### Description of male holotype

(Figs 2–5). Body and eyes pigmented. Eyes not reduced. Body subovate, slightly compressed laterally. Pleon narrower then cephalothorax (width 12.9 mm and 13.6 mm, respectively). Rostrum (Figure 3A) broad in shape, reaching nearly to end of ultimate antennular peduncle more than three times longer than wide (width 3.3 mm at base, length 10,9 mm). Upper surface smooth, short scattered hairs present. Margins slightly elevated continuing in rostral carinae on carapace, almost straight in basal part, distally rather moderately tapering towards apex. Scattered, short, fine hairs present on rostrum surface. Lateral rostral margin bearing three prominent teeth in distal half on right side and two on left, pointing upwards at angle of approximately 45°. Few short hairs present on distal half of outer margins. Acumen with anteriorly orientated spine.

*Rostral carinae* extending as slight elevation posteriorly on carapace terminating at ending of postorbital ridges. Postorbital ridges well developed, terminating in spiniform tubercle anteriorly, fading at two-thirds of occipital carapace length, posteriorly. (Figure 4) Dorsal surface of carapace smooth, scattered fine short hairs present after cervical grove. Cervical and branchiocardiac grooves distinct, setose at middle part, three prominent corneous spine and two smaller spines present at middle part behind cervical groove on lateral sides of carapace.

*Areola* length 9.3 mm, narrowest width 5.1mm. Length of areola 31.8% of total length of carapace (31.17 mm).

Ventrolateral parts smooth with scattered short hairs; anterior margin strongly produced, rounded upper margin directed inward.

*Scaphocerite* broadest at midlength, convex in distal part becoming narrower in basal part; thickened lateral margin terminating in large corneous spine, almost reaching distal margin of ultimate segment of antennular peduncle. Right scaphocerite 7.7 mm long and 2.7 mm wide. Proximal margins setose. Antennulae and antennae typical for genus. Antennae approx. 10 % longer then body. Antennular peduncle reaching slightly behind acumen, antennal peduncle reaching slightly behind apex of scaphocerite. Antennal protopodite with spine anteriorly; basicerite with one lateral and one ventral spine.

*Mouthparts* typical for the genus. Epistome with subcordiform cephalic lobe anteriorly bearing lanceolate cephalomedian projection constricted at base. Lateral margins of lobe not thickened; each lateral margin with two groups of 5-6 tubercles separated by a smooth place. Central part smooth, not pitted, excavate. Eyes rather large; cornea globular, darkly pigmented, nearly as long as eyestalk; eyestalk slightly narrower than cornea.

*First pereopod* equal in form, chela slightly gaping, equal in size, right cheliped (24.6 mm long, 5.2 mm high, 11.2 mm wide). Right chelae (Figure 3B, C), strongly compressed. Fingers shorter than palm (dactylus 10.4 mm long). Dactylus broad at base (3.5 mm), tapering slightly towards tip. Tip with sharp, corneous, hooked tooth pointing outwards at an angle of 45°. Cutting edge of dactyl with continuous row of rather small granular teeth and one prominent larger tooth at middle of cutting edge. Ventral and dorsal surface of movable finger with scattered punctuation. Fixed finger triangular, merging gradually into palm, ending in sharp, corneous, hooked tooth, standing almost perpendicular to axis of finger. Tips of fingers slightly crossing when fingers clasp. Upper surface of palm practically smooth, slightly pitted, more densely pitted at margins. Fixed finger with approximately same width as dactyl at base (5.0 mm). Dense, short setae present in posterior ventral part of fixed finger. Cutting edge of fixed finger with row of rather small granular teeth at posterior half and one at middle of anterior part. Scattered, short hairs present in posterior ventral half of dactylus.

Dorsal surface of *carpus* (8.7 mm) smooth and pitted, with slight excavation in middle part and with a well-developed mesial carpal spine. Ventral carpal surface margins slightly elevated, setose at inner margin and with fovea; inner margin with well-developed ventral carpal spine and ventromesial carpal spine oriented in angle of approx. 45°. Outer lateral margin of chelae with swollen soft and uncalcified patch (7.3 mm) which extends from approx. first third of palm to approx. beginning of the movable finger. Mesial margin of palm slightly elevated, forming slender serrated ridge with row of 10-11 small granular teeth (Figure 5A, B).

*Merus* (13 mm) laterally depressed in basal part; surface slightly pitted; small dorsal meral spine present. Inner ventrolateral margin densely covered with small granules, four sharp ventral meral spines present, one at midlength, other in middle of anterior part, third and fourth on distal ventrolateral inner margin.

*Ischium* (7.8 mm) smooth with two small spines and two granules at midlength of ventrolateral inner margin.

*Second pereopod* reaching anteriorly to approximately corneus spine of scaphocerite. Finger (3.5 mm) slightly shorter as palm (4.0mm), of same height. Scattered short setae present on dactyl and fixed finger. Cutting edge of fixed finger and carpus with row of dense, short setae. Carpus (5.4 mm), smooth, slightly pitted, longer than palm. Merus (9.5 mm) 1.75 times longer than carpus. Ischium (4.6 mm) approx. as half as long as merus.

*Third pereopod* overreaching second by length of finger of second pereopods. Fingers shorter than palm.

*Fourth pereopod* reaching distal margin of scaphocerite. Dactylus with corneous tip. Short scattered setae present. Propodus more than twice as long as dactylus, nearly 1.5 times as long as carpus; somewhat flattened, carrying many stiff setae on lower margin. Merus just slightly longer than propodus.

*Fifth pereopod* similar to fourth, slightly shorter.

Dorsal surface of pleon smooth, with scattered pits; abdominal segments with short setae present on caudal margins.

*Telson* with posterolateral spines, dense short setae present in posterior third. Posterior margins setose. Uropodal protopod with two distal spines on mesial lobe. Exopod of uropod with transverse row of posteriorly directed diminutive spines ending in one more prominent spine, posteriorly directed on outer margin of mesial lobe. Terminal half of exopod with small tubercles and short hairs, slightly corrugated. Endopod of uropod smooth. Short scattered hairs present on posterior third of dorsal exopod. Postrolateral spine on outer margin present. Second spine on medial dorsal surface present, directed posteriorly.

###### Description of allotype female

**Figure 6.**
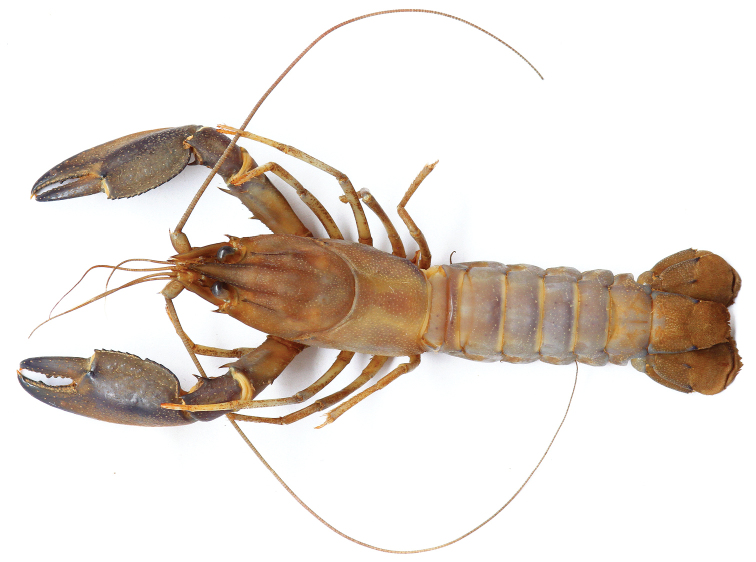
*Cherax mosessalossa* sp. n., allotype female (MZB Cru 4676).

(Figure 6). Chela of first pereopods equal, 2.6 times as long as broad (28.6 mm and 10.9 mm respectively). Mesial margin of palm slightly elevated, forming slender serrated ridge with row of 12-13 small granular teeth. Cutting edge of dactylus with 4-5 rather small granular teeth. Cutting edge of fixed finger with 84-5 small granules. Small scattered short setae visible along ventral cutting edge of chelae, denser and long in ventral posterior area. Tips of fingers slightly crossing when fingers clasp, not gaping. Cervical groove distinct, short setae present at middle part. One prominent corneous spine and two smaller granules present at middle part behind cervical groove on lateral sides of carapace. Pleon just slightly narrower than cephalothorax (widths 12 mm and 12.5 mm respectively). Same colour pattern as in males, less intense.

**Size.** The largest male examined is the holotype with a carapace length of 31.3 mm, and a total length of 79 mm; the paratype male has a total length of 66.3 mm and the other male has a total length of 59 mm; the allotype female has a carapace length of 40 mm and a total length of 87.7 mm (n = 6).

**Colour.** The living animals (Figure 1A, B) are coloured as follows. Male: Chelae dark blue with white margins and white patch. Anterior part usually dark blue. Corneous tooth on tip of fingers orange. Cephalothorax brown-black, with creamy spots laterally. Dark reddish patch on dorsolateral side of the carapace between rostral carinae and cervical grove. Segments of pleon dark blue or brown with yellowish cream transversal band. Lateral pleura slightly lighter with some yellowish cream spots. Walking legs dark bluish grey. Distal margin of tail-fan creamy orange to light yellowish. Females: usually same colour as males somewhat less colourful.

###### Molecular phylogenetic results.

*Cherax mosessalossa* sp. n. is sister species to *Cherax misolicus* (16S only, Figure 19), both are in turn sister group to *C. warsamsonicus*. *Cherax mosessalossa* sp. n. is well isolated from both *C. misolicus* with a sequence divergence (p-distance, 16S) of 1.8 % and from *C. warsamsonicus* with a sequence divergence of 2.6 % (16S) and 6.6–7.0 % (COI), respectively, supporting the morphology-based description of *C. mosessalossa* as a new species.

###### Systematic remarks.

Among all species of the northern group, *C. mosessalossa* sp. n. is most similar to *C. misolicus*, a species that is endemic to Misool Island, one of four major islands in the Raja Ampat Islands in West Papua, Indonesia, and to C. *warsamsonicus*, a species that is endemic to the Warsamson River ca. 50 km north of Sorong. *Cherax mosessalossa* sp. n. differs from *C. misolicus* and *C. warsamsonicus* in several characters(see Table 2).

###### Etymology.

*Cherax mosessalossa* sp. n. is named after the son of our guide Marten Luther Salossa, Moses Yorof Salossa, who died of malaria at the age of 2.

###### Ecology.

Known only from the Klademak Creek, South Sorong Regency, in the central part of the Kepala Burung (Vogelkop) peninsula. (Figure 8) At the sampling site the creek is shallow (20–50 cm) with a moderate flow and had a pH of approximately 6.5. In most parts no water plants are present. The substrate of the creek is gravel or sand and soil mostly covered with silt and detritus, stones and larger rocks (Figure 9). Crayfish hide in short borrows in the creek bank, under lager rocks or in detritus that gathers in slower flowing parts of the creek. To improve the knowledge of the ecology and distribution of the species more field surveys will be necessary.

###### Common name.

As common name for this crayfish we propose Klademak Creek Crayfish.

**Figure 7.**
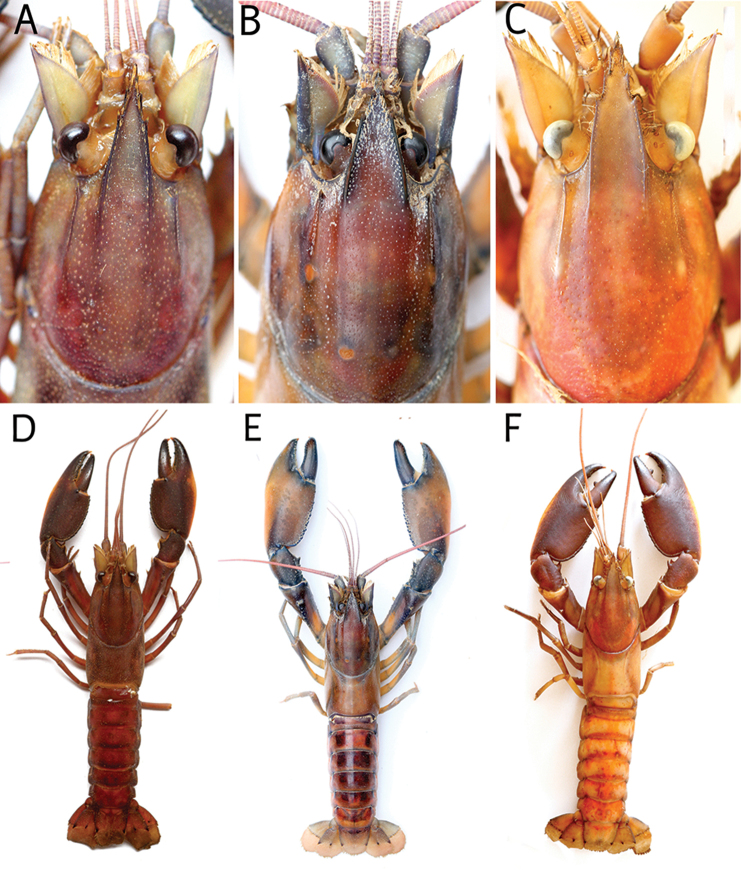
Rostrum dorsal view **A** *Cherax mosessalossa* sp. n. holotype male (MZB Cru 4675) **B** *Cherax misolicus* (this study) **C** *Cherax warsamsonicus*, holotype male, (MZB Cru 4529)Dorsal view **D** *Cherax mosessalossa* sp. n. holotype male (MZB Cru 4675) . **E** *Cherax misolicus* (this Study) **F** *Cherax warsamsonicus*, holotype male, (MZB Cru 4529)

**Figure 8.**
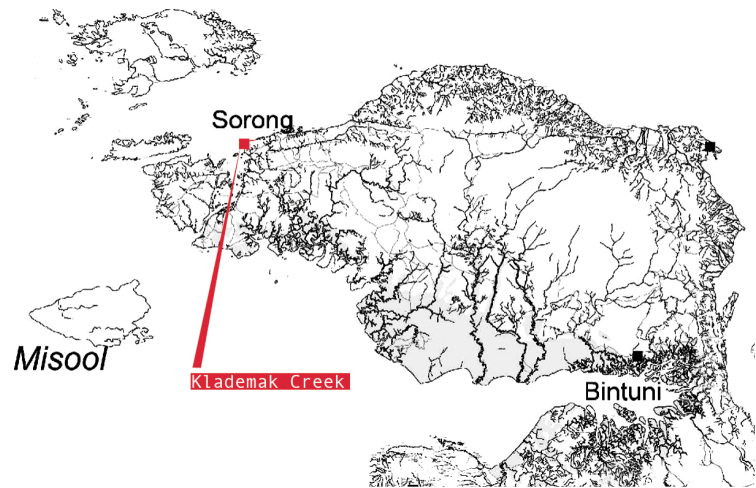
The Bird’s Head Peninsula, West Papua, Indonesia with the type locality, Klademak Creek, indicated.

**Figure 9.**
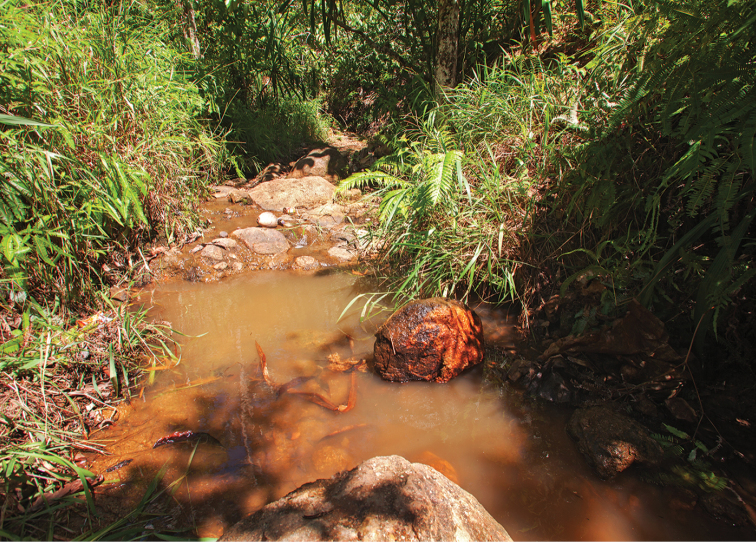
Klademak Creek, habitat of the new species.

**Table 2.** Comparison of morphological features of *C. mosesalossa* sp. n., *C. misolicus*, and *C. warsamsonicus*.

	*C. mosesalossa* sp. n.	*C. misolicus*	*C. warsamsonicus*
Chelae	4.5–4.7 times as long as height, 2.0–2.1 times as broad as height.	4.0–4.2 times as long as height, 1.6–1.7 times as broad as height.	4.6 times as long as height, 2.3 times as broad as height
Rostrum	3 times as long as broad, rather straight	2 times as long as broad, rather straight	2.5 times as long as broad, clearly bent outwards at middle part.
Lateral sides of the carapace	3 prominent corneous spines, 2 smaller spines on middle part behind cervical groove	6–7 very small tubercles on middle part behind cervical groove	1 prominent corneous spine and 3 tubercles on middle part behind cervical groove
Setae	Rostrum: less setose, Chelae: setae on the ventral side of the cutting edges of the chelae, Carpus: setae present on the inner margin Carapax :short fine hairs present.	Rostrum: setose Chelae: ventral side with short setae on edge of fixed finger and movable finger. Carapax;fine short hairs present.	Rostrum: non setose,few bristles.Chelae: ventral side of cutting edged not setose,few short bristles Carapax: non setose.
Coloration of the chelae	dark grey blue, whiter at the margins, with a white patch.	light blue with yellow creamy margins and a white patch.	dark blue chelae , white coloured lateral margin, white patch.
Coloration of the body	dark brown with white spots.	creamy to brown and yellow.	greenish grey with red to pink patches dorsally.
Coloration of the cephalothorax	brown grey with creamy spots laterally.	creamy to yellow.	greenish black, with small slightly darker spots laterally and ventrally fading to grey-green.
Coloration of the segments of the pleon	dark blue or brown with cream yellowish transversal bands.	creamy to yellow,light green dorsally with a black pattern.	with pink red band anteriorly becoming black.

##### Cherax alyciae sp. n.

Taxon classificationAnimaliaDecapodaParastacidae

http://zoobank.org/A18FE24A-8259-4B98-9CE4-C5112C47E6A5

**Figure 10.**
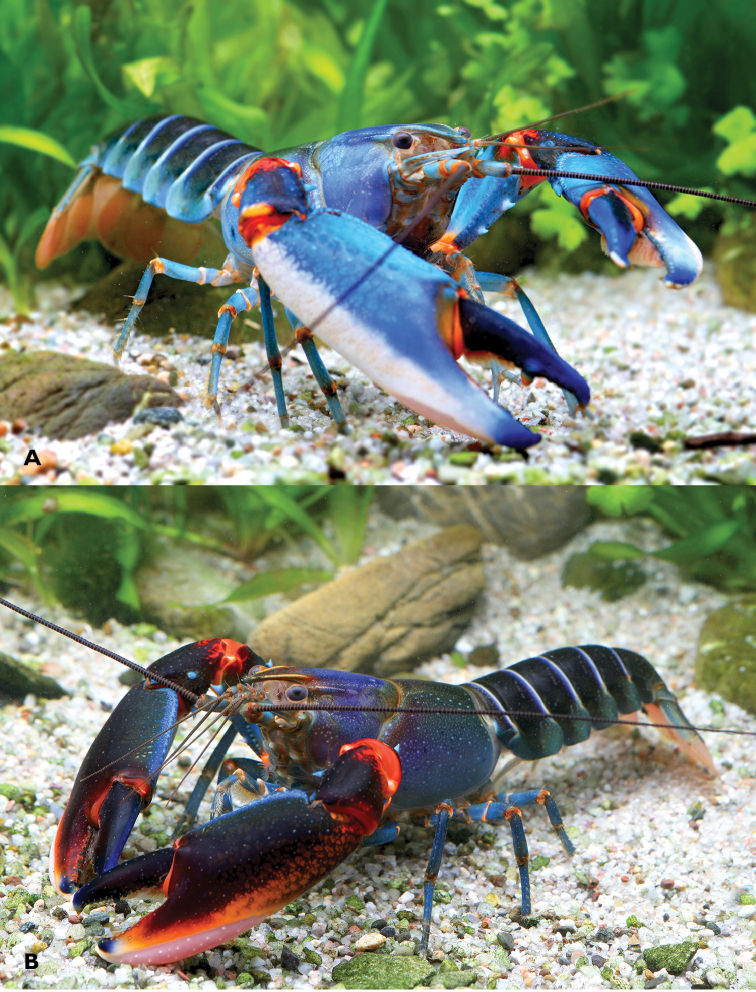
*C. alyciae* sp. n. **A** holotype male (MZB Cru 4672) from nameless creek, Boven Digoel Regency **B** idem, colour variation.

**Figure 11.**
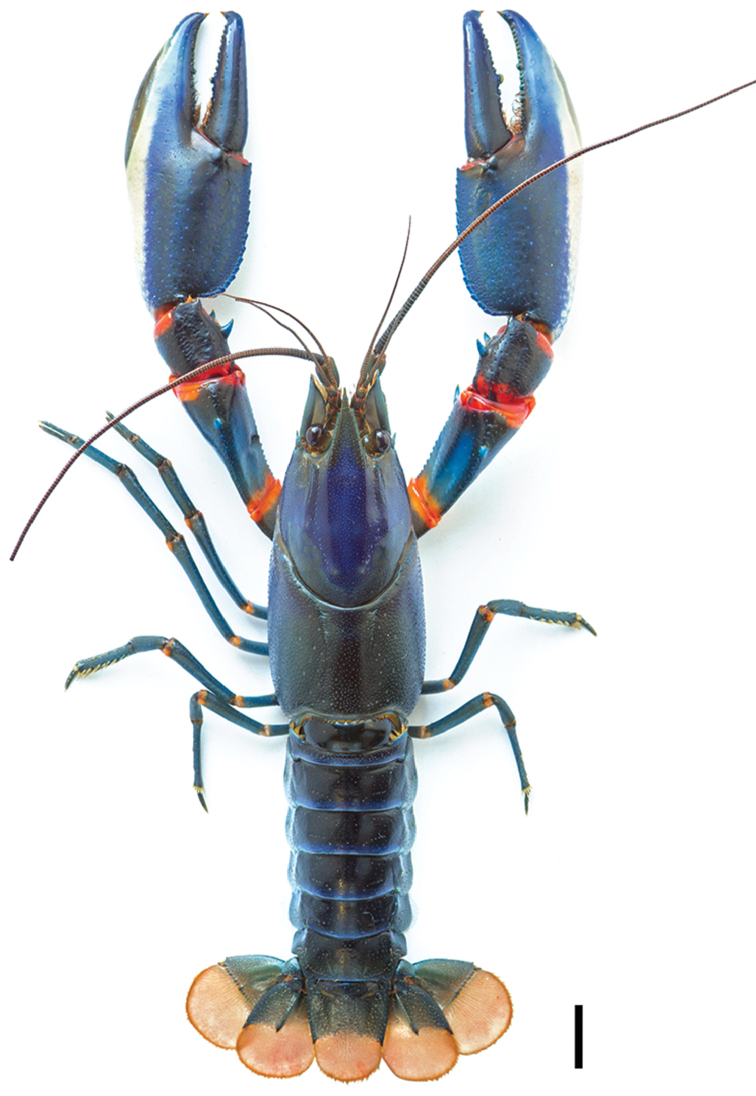
*C. alyciae* sp. n. holotype male (MZB Cru 4672). Scale bar: 10 mm.

**Figure 12.**
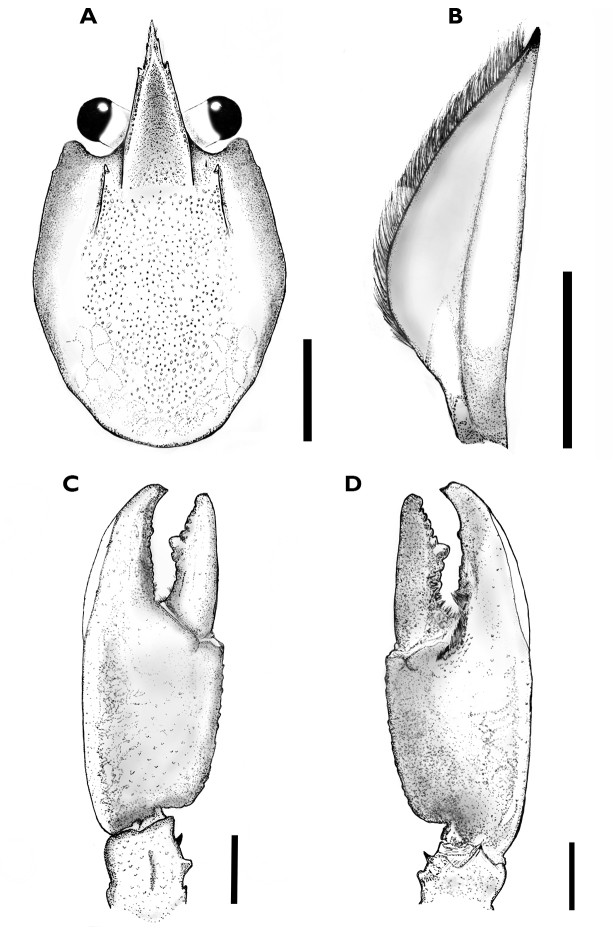
*C. alyciae* sp. n. holotype male (MZB Cru 4672) **A** dorsal view of carapace **B** scaphocerite **C** dorsal view of left chelae **D** ventral view of left chelae. Scale bars: 10 mm (**A, C, D**), 5 mm (**B**).

**Figure 13.**
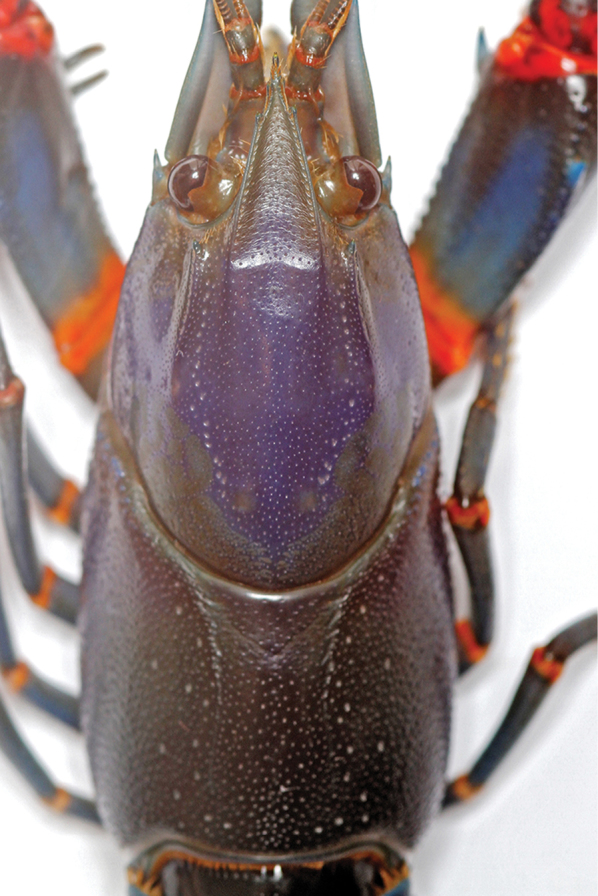
*C. alyciae* sp. n. holotype male (MZB Cru 4672), dorsal view of cephalothorax. Scale bar: 10 mm.

**Figure 14.**
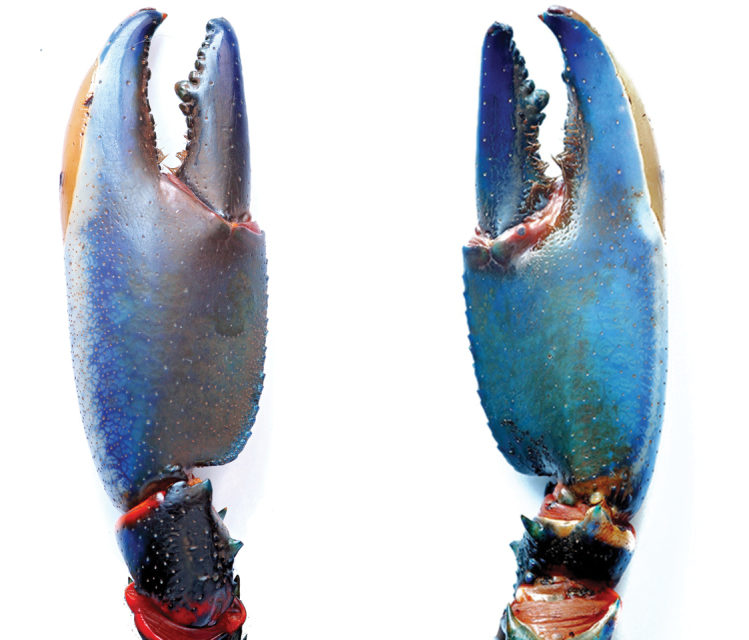
*C. alyciae* sp. n. holotype male (MZB Cru 4672) **A** left first chela, dorsal aspect **B** left first chela, ventral aspect. Scale bars: 10 mm.

Figs 10 , 11 , 12 , 13 , 14

###### Material examined.

**Holotype**: male (MZB Cru 4672), under rocks, among roots and in debris along banks of a nameless creek, Boven Digoel Regency, West Papua, Indonesia. coll. local catchers and fisherman, December 7, 2016. **Allotype**: female (MZB Cru 4673), same data as holotype. **Paratypes**: (MZB Cru 4674) and (ZMB 30708), same data as holotype. Exact location stored with type material to protect population in its natural habitat.

###### Diagnosis.

Carapace surface covered with small granules, areola pitted, no spines present posterior to cervical groove on lateral carapace. Eyes large, pigmented. Cornea slightly broader than eyestalk. Rostrum triangular in shape with elevated margins, setose in the anterior marginal half. Rostral margins with two prominent teeth, rostral carinae prominent. Postorbital ridges prominent with one acute tubercle at anterior terminus. Carapace blue, dorsal usually darker blue to green. Uncalcified patch on lateral margin of chelae of adult male white, translucent. Propodal cutting edge with row of small granules and one larger tubercle. Chelipeds blue, lateral margins white, posterior lateral part sometimes orange. Fingers blue, in posterior third dark blue with hooked orange tips. Other walking legs blue-green with orange joints. Pleon dark blue with light blue transverse lines.

###### Description of male holotype

(Figs 11–14). Body and eyes pigmented. Eyes not reduced. Body subovate, slightly compressed laterally. Pleon narrower then cephalothorax (width 21.8 mm and 24.9 mm respectively). Rostrum (Figure 12A) broad in shape, reaching nearly to end of ultimate antennular peduncle and approx. twice as long than wide (width 6.1 mm at base, length 11.7 mm). Margins slightly elevated continuing in rostral carinae on carapace, almost straight in basal part, distally rather moderately tapering towards apex. Lateral rostral margin bearing two prominent teeth in distal half, pointing upwards at angle of approximately 45°. Few short hairs present on distal half of outer margins between the first teeth and the acumen. Acumen with anteriorly orientated spine.

Rostral carinae extending as slight elevation posteriorly on carapace terminating at ending of postorbital ridges. Postorbital ridges well developed, terminating in spiniform tubercle anteriorly, fading at two-thirds of occipital carapace length, posteriorly. Postorbital ridges approx. 1/3 of CL. Cervical and branchiocardiac grooves distinct, non-setose, six tiny and weak developed tubercles present at middle part behind cervical groove on lateral sides of carapace. Carapace surface densely covered with tiny granules, anterior margin strongly produced, rounded upper margin directed inward.

Areola length 18.4 mm, narrowest width 8.0 mm. Length of areola 34.7% of total length of carapace (54 mm). Densely pitted.

*Scaphocerite* (Figure 12B), broadest at posterior third, convex in distal part becoming narrower in basal part; thickened lateral margin terminating in corneous spine, almost reaching distal margin of ultimate segment of antennular peduncle. Right scaphocerite 12.0 mm long and 3.9 mm wide. Proximal margins setose. Antennulae and antennae typical for genus. Antennae slightly longer then body. Antennular peduncle reaching slightly behind acumen, antennal peduncle reaching slightly behind apex of scaphocerite. Antennal protopodite with spine anteriorly; basicerite with one lateral and one ventral spine.

*Mouthparts* typical for the genus. Epistome with sub-cordiform cephalic lobe anteriorly bearing lanceolate cephalomedian projection constricted at base. Lateral margins of lobe not thickened; each lateral margin with two groups of 8-9 tubercles separated by a smooth place. Central part smooth, not pitted, excavate. Eyes rather large; cornea globular, darkly pigmented, nearly as long as eyestalk; eyestalk slightly narrower than cornea.

*First pereopod* equal in form, chela slightly gaping. Right cheliped 56 mm long, 12 mm high, 21 mm wide. Left chelae (Figure 12C, D) 51.8 mm long and 10.4 mm high, 20.6 mm wide, strongly compressed. Fingers shorter than palm (right dactylus 25.4 mm long). Dactylus broad at base (7.8 mm), tapering slightly towards tip.

Tip with sharp, corneous, hooked tooth pointing outwards at an angle of 45°. Cutting edge of dactyl with continuous row of rather small granular teeth and one prominent larger tooth at middle of cutting edge. Ventral and dorsal surface of movable finger with scattered punctuation. Ventral posterior half of cutting edge with with dense setae reaching from base to prominent larger tooth. Fixed finger triangular, merging gradually into palm, ending in sharp, corneous, hooked tooth, standing almost perpendicular to axis of finger. Tips of fingers slightly crossing when fingers clasp. Upper surface of palm practically smooth, slightly pitted, more densely pitted at margins. Fixed finger slightly broader than dactyl at base (11.3 mm). Dense, short setae present in posterior ventral part of fixed finger, reaching from base to midlength. Cutting edge of fixed finger with row of rather small granular teeth at posterior half and one bigger one at midlength. Outer lateral margin of chelae with swollen soft and uncalcified patch (23 mm) which extends from about middle of palm to midlength of opposable dactylus. Row of 20-21 mesial probodal granules at dorsolateral margin. Dorsolateral margins slightly elevated.

Dorsal surface of *carpus* (14.4 mm) smooth and pitted, with slight excavation in middle part and with a well-developed mesial carpal spine. Ventral carpal surface margins slightly elevated, non-setose and with fovea; inner margin with well-developed ventral carpal spine and ventromesial carpal spine oriented in angle of approx. 45°.

*Merus* (24.7 mm) laterally depressed in basal part; surface slightly pitted; small dorsal meral spine present. Inner ventrolateral margin densely covered with small granules, three ventral meral spines present, one at midlength other in middle of anterior part, third on distal ventrolateral inner margin.

*Ischium* (14.69 mm) smooth with two small spines and eleven granules at midlength of ventrolateral inner margin.

*Second pereopod* reaching anteriorly to approximately corneus spine of scaphocerite. Finger (7.0 mm) slightly longer as palm (6.6mm), of same height. Scattered short setae present on dactyl and fixed finger. Cutting edge of fixed finger and carpus with row of dense, short setae. Carpus (9.3 mm), smooth, slightly pitted, longer than palm. Merus (17.4 mm) 1.87 times longer than carpus. Ischium (8.3 mm) about as half as long as merus.

*Third pereopod* overreaching second by length of finger of second pereopods. Fingers shorter than palm.

*Fourth pereopod* reaching distal margin of scaphocerite. Dactylus with corneous tip. Short scattered setae present. Propodus more than twice as long as dactylus, nearly 1.5 times as long as carpus; somewhat flattened, carrying many stiff setae on lower margin. Merus just slightly longer than propodus.

*Fifth pereopod* similar to fourth, slightly shorter.

Dorsal surface of pleon smooth, with scattered pits; abdominal segments with short setae present on caudal margins.

*Telson* with posterolateral spines, dense short setae present in posterior third. Posterior margins setose. Uropodal protopod with two distal spines on mesial lobe. Exopod of uropod with transverse row of posteriorly directed diminutive spines ending in one more prominent spine, posteriorly directed on outer margin of mesial lobe. Terminal half of exopod with small tubercles and short hairs, slightly corrugated. Endopod of uropod smooth. Short scattered hairs present on posterior third of dorsal exopod. Posterolateral spine on outer margin present. Second spine on medial dorsal surface present, directed posteriorly.

###### Description of allotype female

**Figure 15.**
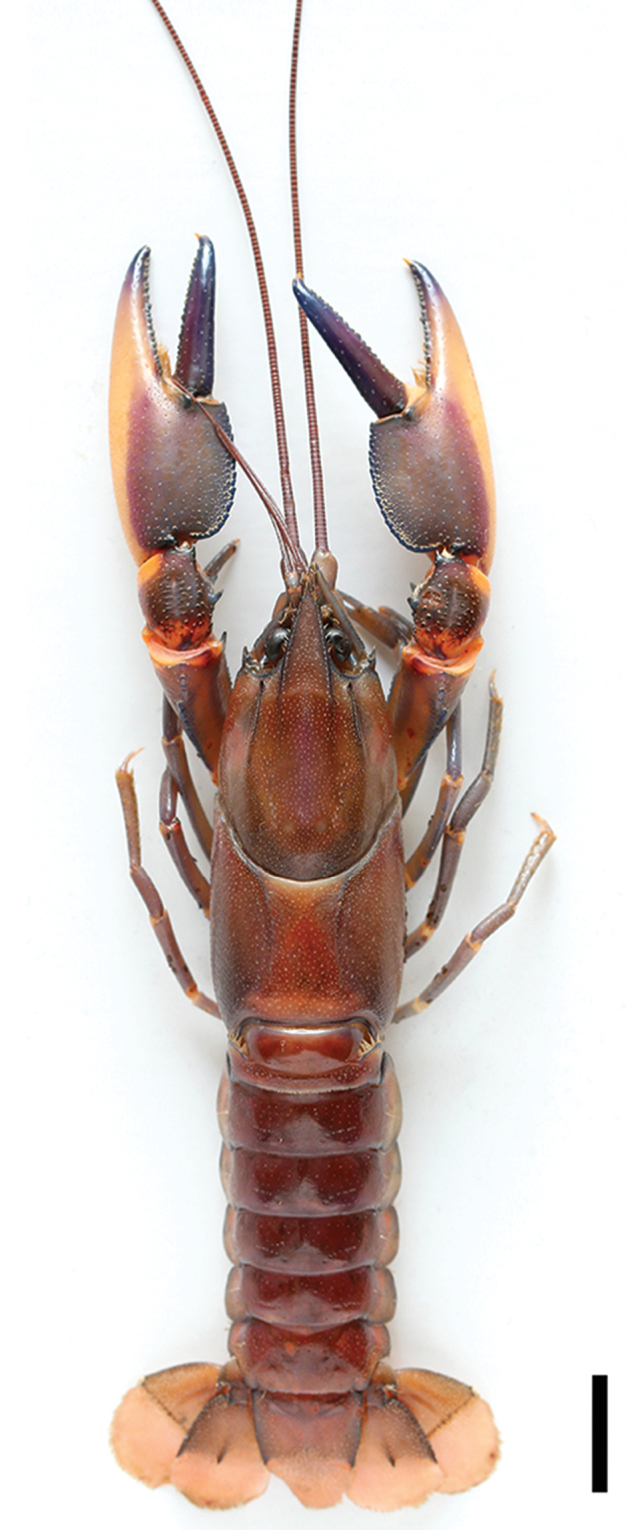
*C. alyciae* sp. n. allotype female (MZB Cru 4673). Scale bar: 10 mm.

(Figure 15). Chela of first pereopods equal, 2.7 times as long as broad (30.6 mm and 11.2 mm respectively). Mesial margin of palm slightly elevated, forming slender serrated ridge with row of 24–25 small granular teeth. Cutting edge of dactylus with 16-17 rather small granular teeth. Cutting edge of fixed finger with 16 small granules. Small scattered short setae visible along ventral cutting edges of chelae, denser and long in ventral posterior area. Tips of fingers slightly crossing when fingers clasp, not gaping. Cervical groove distinct, non-setose. Pleon just slightly narrower than cephalothorax (widths 16.2mm and 16.6 mm respectively). Same colour pattern as in males. No soft patch in females observed (n = 120).

*Size.* The largest male examined has a carapace length of 56 mm and a total length of 122 mm; the holotype male has a total length of 117 mm; the other males have a total length between 78 mm and 119 mm; the allotype has a carapace length of 39 mm and a total length of 86 mm (n = 11).

*Colour.* The living animals (Figure 1A, B) are coloured as follows. Male: Chelae light to dark blue with white margins and white patch. Anterior part usually dark blue, more intense coloured. Corneous tooth on tip of fingers orange. Cephalothorax bright blue, dorsally more intense from purple to greenish blue, fading ventrally to light blue. Joints between propodus and carpus and between carpus and merus bright orange-red. Segments of pleon dark blue to black, lateral pleura lighter becoming blueish green. Light blue transverse bands in the posterior part of each pleonary somite. Walking legs light blue with orange joints. Distal margin of tail-fan creamy orange to orange. Some animals are darker and differ in the colouration of chelae. Chelae dark blue to black, becoming orange-red at the outer lateral margin. Dorsolateral margins light blue. These males have usually also orange or yellow rostral margins. Females: same colour as males, sometimes less intense.

###### Molecular phylogenetic results.

*Cherax alyciae* sp. n. is sister species to *Cherax peknyi* (Figure 19), both are in turn sister group to *C. warsamsonicus*. *Cherax alyciae* sp. n. is well isolated from *C. peknyi* with a sequence divergence (p-distance) of 2.1–2.8 % (16S) and 5.7–6.3 % (COI), respectively, supporting the morphology-based description of *C. alyciae* as a new species.

###### Deposition of types.

The holotype (MZB Cru 4672), allotype (MZB Cru 4673) and paratypes (MZB Cru 4674) are deposited at the Museum Zoologicum Bogoriense (= Bidang Zoologi) Reseach Centre for Biology (= Pusat Penelitian Biologi), Indonesian Institute of Sciences (= LIPI), Jalan Raya Jakarta-Bogor Km 46 Cibinong 16911, Indonesia. Additional Paratypes are deposited at the Museum für Naturkunde, Leibniz Institute for Evolution and Biodiversity Science, Berlin (ZMB 30708).

###### Systematic position.

Holthuis (1949) in his publication on the New Guinea *Cherax* considered species should be placed into two groups. One with the rostral and median carinae absent or weakly developed and referred to as the *Cherax* group following the characteristics of the type species, *C. preissii* (Erichson, 1846) from southwest Australia. The other group contains species that have the rostral and sometimes the median carina well developed and referred to as the *Astaconephrops* group with Nobili’s (1899) *Astaconephrops albertisii* as the type. Newly described species have been placed into one or the other of the two subgenera (Lukhaup and Pekny 2006; Lukhaup and Pekny 2008; Lukhaup and Herbert 2008; Lukhaup 2015, Lukhaup et al. 2015; Patoka et al. 2015). Munasinghe et al. (2004a, b), Austin (1996); and Austin et al. (1996) however, identified three lineages with different geographic ranges within *Cherax* based on molecular genetics and phylogenetic studies. These consist of a southwestern group, an eastern group and a northern group. Support for the latter group, however, was based on only very limited sampling (e.g., single samples of *C. quadricarinatus*, *C. rhynchotus*, and *C. peknyi* in Munasinghe et al. (2003). Munasinghe et al. (2004b) indicate that the division of *Cherax* into two subgenera, as conceived by Holthuis and subsequent authors dealing with New Guinea crayfish, has to be reconsidered. Based on Munasinghe et al. (2004a, b), Austin (1996), and Austin et al. (1996), *Cherax warsamsonicus* sp. n. and *Cherax alyciae* sp. n. belong to the northern species group lineage consisting of 25 species: *C. acherontis* Patoka, Bláha & Kouba, 2017,*C. albertisii* (Nobili, 1899), *C. alyciae* sp. n. (this study),*C. boesemani* Lukhaup & Pekny, 2008, *C. boschmai* Holthuis, 1949, *C. buitendijkae* Holthuis, 1949, *C. communis* Holthuis, 1949, *C. gherardii* Patoka, Bláha & Kouba, 2015, *C. holthuisi* Lukhaup & Pekny, 2006, *C. lorentzi aruanus* (Roux, 1911), *C. lorentzi lorentzi* (Roux, 1911), *C. longipes* Holthuis, 1949, *C. misolicus* Holthuis, 1949, *C. murido* Holthuis, 1949, *C. monticola* Holthuis, 1950, *C. mosessalossa* sp. n. (this study), *C. minor* Holthuis, 1996, *C. peknyi* Lukhaup & Herbert, 2008, *C. pallidus* Holthuis, 1949, *C. papuanus* Holthuis, 1949, *C. paniaicus* Holthuis, 1949, *C. pulcher* Lukhaup, 2015, *C. solus* Holthuis, 1949, *C. snowden* Lukhaup, Panteleit & Schrimpf, 2015, and *C. warsamsonicus* Lukhaup, Eprilurahman & von Rintelen, 2017.

###### Systematic remarks.

**Figure 16.**
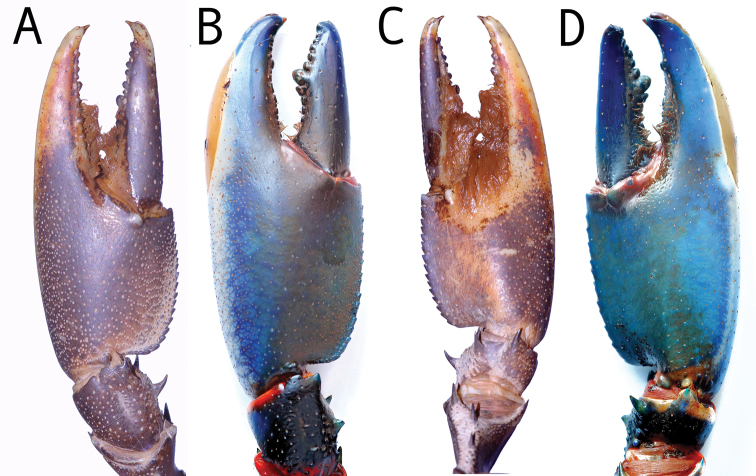
Dorsal view chelae. **A** *Cherax peknyi*, holotype male (QMW28267) **B** *C. alyciae* sp. n. holotype male (MZB Cru 4672) ventral view chelae **C** *Cherax peknyi*, holotype male, (QMW28267) **D** *C. alyciae* sp. n. holotype male (MZB Cru 4672).

In comparison to all species of the northern group the new species, *C. alyciae* sp. n. is most similar to *C. peknyi*, a species that is known from the Fly River drainage, close to the City of Kiunga, Papua New Guinea. *Cherax alyciae* sp. n. differs from *C. peknyi* in the following characters: shape of the chelae (Figure 16A–D), the presence of a soft patch in the chelae of males and in colouration. The rostrum of *Cherax alyciae* sp. n. is rather straight, triangular shaped, while the rostrum of *Cherax peknyi* is clearly bent outwards at the middle part. *Cherax peknyi* has 3–4 anteriorly directed spines present at middle part behind cervical groove on lateral sides of carapace while *C. alyciae* sp. n. has six tiny and weak developed tubercles there. *Cherax peknyi* has usually red to orange chelae becoming white posteriorly with blue fingertips, the carapace is orange yellow becoming dark red dorsally while the pleon is dark green with orange yellow transverse bands. None of the males of *C. peknyi* had a soft patch. Furthermore, the presence of dense setae on the ventral chelae in *C. peknyi* while *C. alyciae* sp. n. has just very few setae there. *Cherax peknyi* is endemic in the Fly River drainage in Papua New Guinea, while *C. alyciae* sp. n. is found in creeks and rivers of the Digul River drainage in the eastern part of the Boven Digoel Regency, Papua, Indonesia.

###### Etymology.

*Cherax alyciae* sp. n. is named after Alycia Evanya, the daughter of Christian Jeffrey (Maju Aquarium) who brought the species to our attention.

###### Ecology.

**Figure 17.**
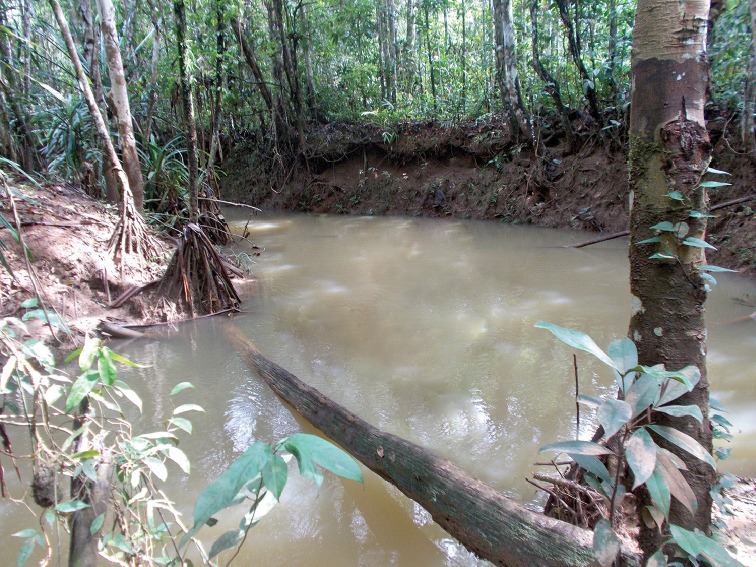
Unnamed Creek, Boven Digoel Regency, habitat of the new species.

Known only from several nameless creeks in the Boven Digoel Regency in the eastern Part of Papua Province, Indonesia, close to the border of Papua New Guinea. The creek harbouring these crayfish is shallow (20–70 cm) with a moderate flow and had a pH of approximately 5.0. The temperature is around 25–27 °C and 12 µS/cm. In most parts no water plants are present. The substrate of the creek is gravel or sand and soil mostly covered with silt and detritus, stones and larger rocks (Figure 17). Crayfish hide in short borrows in the riverbank, under larger rocks or in detritus that gathers in slower flowing parts of the creek. To improve the knowledge of the distribution of the species more field surveys will be necessary.

###### Common name.

As common name for this crayfish we propose Blue Kong Crayfish as it is already known under this name in the pet trade.

**Figure 18.**
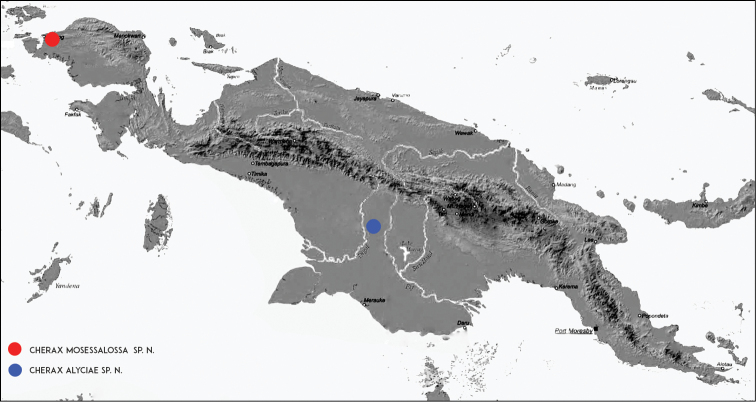
Map of Papua New Guinea, with the type localities indicated.

**Figure 19.**
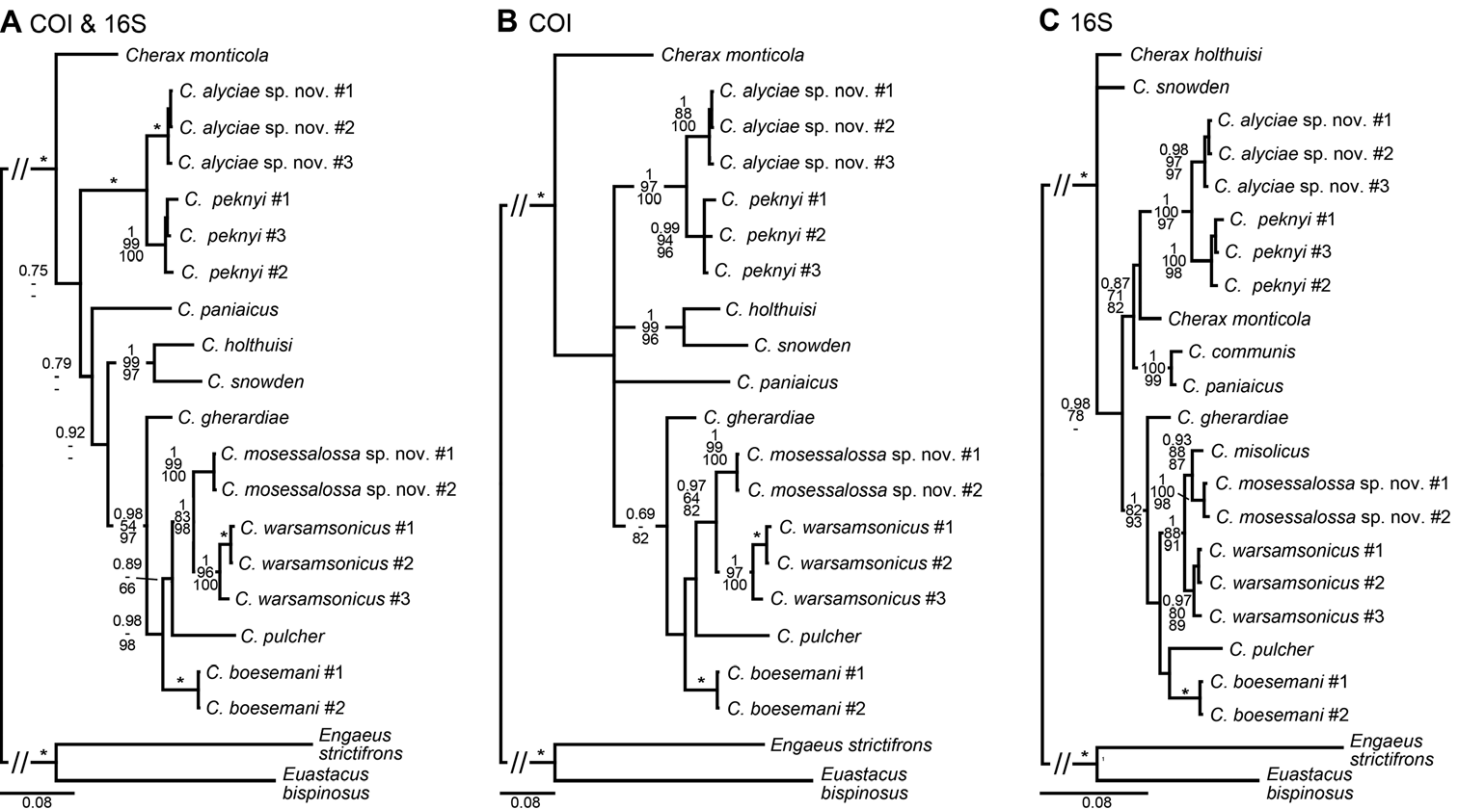
Phylogenetic relationships of *Cherax mosessalossa* sp. n. and *C. alyciae* sp. n. within the northern New Guinea *Cherax* lineage, reconstructed by BI analyses of two mitochondrial gene fragments. Number on branches show, from top, Bayesian posterior probabilities (>0.7) and ML/MP bootstrap values (>50). An asterisk indicates nodes with full support (1/100/100) in all analyses. The scale bar indicates the substitution rate. See Table 1 for information on the sequenced specimens. **A** Topology based on concatenated COI and 16S dataset **B** Topology based on COI dataset **C** Topology based on 16S dataset.

## Supplementary Material

XML Treatment for Cherax mosessalossa

XML Treatment for Cherax alyciae

## References

[B1] Austin CM (1996) Systematics of the freshwater crayfish genus *Cherax* Erichson (Decapoda: Parastacidae) in northern and eastern Australia: electrophoretic and morphological variation. Australian Journal of Zoology 44: 259–296. https://doi.org/10.1071/ZO9960259

[B2] Austin CM Knott B (1996) Systematics of the freshwater crayfish genus *Cherax* Erichson (Decapoda: Parastacidae) in south-western Australia: electrophoretic, morphological and habitat variation. Australian Journal of Zoology 44: 223–258. https://doi.org/10.1071/ZO9960223

[B3] Crandall KA Fitzpatrick JF (1996) Crayfish molecular systematics: using a combination of procedures to estimate phylogeny. Systematic Biology 45: 1–26. https://doi.org/10.1093/sysbio/45.1.1

[B4] Erichson WF (1846) Übersicht der Arten der Gattung *Astacus*. Archiv für Naturgeschichte 12: 86–103, 375–377.

[B5] Folmer O Black M Hoeh W Lutz R Vrijenhoek R (1994) DNA primers for amplification of mitochondrial cytochrome c oxidase subunit I from diverse metazoan invertebrates. Molecular Marine Biology and Biotechnology 3: 294–299. 7881515

[B6] Gan HM Tan MH Eprilurahman R Austin CM (2014) The complete mitogenome of *Cherax monticola* (Crustacea: Decapoda: Parastacidae), a large highland crayfish from New Guinea. Mitochondrial DNA Part A 27: 337–338. https://doi.org/10.3109/19401736.2 014.892105 10.3109/19401736.2014.892105 24617471

[B7] Holthuis LB (1949) Decapoda Macrura with a revision of the New Guinea Parastacidae. Zoological results of the Dutch New Guinea Expedition 1939. No. 3. Nova Guinea (n. ser. ) 5: 289–330.

[B8] Holthuis LB (1956) Native fisheries of freshwater Crustacea in Netherlands New Guinea. Contributions to New Guinea Carcinology. I. Nova Guinea (n. ser. ) 7(2): 123–137. [figs 1–3]

[B9] Holthuis LB (1958) Freshwater crayfish in Netherlands New Guinea Mountains. South Pacific Commission Quarterly Bulletin 8(2): 36–39. [7 figs]

[B10] Holthuis LB (1982) Freshwater Crustacea Decapoda of New Guinea. In: Gressitt JL (Ed.) Biogeography and Ecology of New Guinea (Vol. 2). Monographiae Biologicae 42: 603–619. https://doi.org/10.1007/978-94-009-8632-9_28

[B11] Holthuis LB (1986) The freshwater crayfish of New Guinea. Freshwater Crayfish 6: 48–58.

[B12] Holthuis LB (1996) Cherax (Astaconephrops) minor new species, a parastacid from the mountains of Irian Jaya (W. New Guinea) Indonesia (Crustacea: Decapoda: Parastacidae). Zoologische Mededelingen Leiden 70(24): 361–366.

[B13] Huxley TH (1879) The Crayfish: an Introduction to the Study of Zoology. C. Kegan Paul & Co, London, 371 pp.

[B14] Katoh K Misawa K Kuma K-I Miyata T (2002) MAFFT: a novel method for rapid multiple sequence alignment based on fast Fourier transform. Nucleic Acids Research 30: 3059–3066. https://doi.org/10.1093/nar/gkf436 12136088 10.1093/nar/gkf436 PMC135756

[B15] Kumar S Stecher G Tamura K (2016) MEGA7: Molecular Evolutionary Genetics Analysis version 70 for bigger datasets. Molecular Biology and Evolution 33: 1870–1874. https://doi.org/10.1093/molbev/msw054 27004904 10.1093/molbev/msw054 PMC8210823

[B16] Lukhaup C Pekny R (2006) Cherax (Cherax) holthuisi, a new species of crayfish (Crustacea: Decapoda: Parastacidae) from the centre of the Vogelkop Peninsula in Irian Jaya (West New Guinea), Indonesia. Zoologische Mededelingen Leiden 80–1 (7): 101–107.

[B17] Lukhaup C Pekny R (2008) Cherax (Astaconephrops) boesemani, a new species of crayfish (Crustacea: Decapoda: Parastacidae) from the centre of the Vogelkop Peninsula in Irian Jaya (West New Guinea), Indonesia. Zoologische Mededelingen Leiden 82(33): 331–340.

[B18] Lukhaup C Herbert B (2008) Cherax (Cherax) peknyi sp. nov., a new species of crayfish (Crustacea: Decapoda: Parastacidae) from the Fly River Drainage, Western Province, Papua New Guinea. Memoirs of the Queensland Museum 52(2): 213–219.

[B19] Lukhaup C (2015) Cherax (Astaconephrops) pulcher, a new species of freshwater crayfish (Crustacea, Decapoda, Parastacidae) from the Kepala Burung (Vogelkop) Peninsula, Irian Jaya (West Papua), Indonesia. ZooKeys 502: 1–10. https://doi.org/10.3897/zookeys.502.9800 10.3897/zookeys.502.9800 PMC4443586 26019660

[B20] Lukhaup C Panteleit J Schrimpf A (2015) Cherax (Astaconephrops) snowden a new species of freshwater crayfish (Crustacea, Decapoda, Parastacidae) from the Kepala Burung (Vogelkop) Peninsula, Irian Jaya (West Papua), Indonesia. ZooKeys 518: 1–14. https://doi. org/10.3897/zookeys.518.6127 10.3897/zookeys.518.6127 PMC4591594 26448698

[B21] Lukhaup C Eprilurahman R Rintelen T (2017) *Cherax warsamsonicus*, a new species of crayfish from the Kepala Burung (Vogelkop) peninsula in West Papua, Indonesia (Crustacea, Decapoda, Parastacidae). ZooKeys 660: 151–167. https://doi.org/10.3897/zookeys.660.11847 10.3897/zookeys.660.11847 PMC5549000 28794676

[B22] Munasinghe DHN Burridge CP Austin CM (2004a) The systematics of freshwater crayfish of the genus *Cherax* Erichson (Decapoda: Parastacidae) in eastern Australia re-examined using nucleotide sequences from 12S rRNA and 16S rRNA genes. Invertebrate Systematics 18: 215–225. https://doi.org/10.1071/IS03012

[B23] Munasinghe DHN Burridge CP Austin CM (2004b) Molecular phylogeny and zoogeography of the freshwater crayfish genus *Cherax* Erichson (Decapoda: Parastacidae) in Australia. Biological Journal of the Linnean Society 81: 553–563. https://doi.org/10.1111/j.1095- 8312.2003.00299.x

[B24] Munasinghe DHN Murphy NP Austin CM (2003) Utility of mitochondrial DNA sequences from four gene regions for systematic studies of Australian freshwater crayfish of the genus *Cherax* (Decapoda: Parastacidae). Journal of Crustacean Biology 23: 402–417. https://doi. org/10.1163/20021975-99990350

[B25] Nobili DG (1899) Contribuzioni alia conoscenza della fauna carcinologica della Papuasia. delle Molucche e dell’ Australia. Annali del Museo Civico di Genova, serie 2, 20 (40): 230–282.

[B26] Patoka J Blaha M Kouba A (2015) Cherax (Astaconephrops) gherardii, a new crayfish (Decapoda: Parastacidae) from West Papua, Indonesia. Zootaxa 3964(5): 526–536. https://doi. org/10.11646/zootaxa.3964.5.2 26249463 10.11646/zootaxa.3964.5.2

[B27] Patoka J Bláha M Kouba A (2017) *Cherax acherontis* (Decapoda: Parastacidae), the first cave crayfish from the Southern Hemisphere (Papua Province, Indonesia). Zootaxa 4363(1): 137–144. https://doi.org/10.11646/zootaxa.4363.1.7 29245414 10.11646/zootaxa.4363.1.7

[B28] Posada D (2008) jModelTest: Phylogenetic model averaging. Molecular Biology and Evolution 25: 1253–1256. https://doi.org/10.1093/molbev/msn083 18397919 10.1093/molbev/msn083

[B29] Ronquist F Huelsenbeck JP (2003) MRBAYES 3: Bayesian phylogenetic inference under mixed models. Bioinformatics 19: 1572–1574. https://doi.org/10.1093/bioinformatics/ btg180 12912839 10.1093/bioinformatics/btg180

[B30] Roux J (1911) Nouvelles especes de Decapodes d’eau douce provenant de Papouasie. Notes Leyden Museum 33: 81–106. [figs 1–5]

[B31] Stamatakis A Hoover P Rougemont J (2008) A rapid bootstrap algorithm for the RAxML web servers. Systematic Biology 57: 758–771. https://doi.org/10.1080/10635150802429642 18853362 10.1080/10635150802429642

[B32] Swofford DL (2002) PAUP* (version 4.0). Phylogenetic analysis using parsimony (*and other methods). Sinauer Associates, Sunderland, Mass.

